# Rapid Increase of Genetically Diverse Methicillin-Resistant *Staphylococcus aureus,* Copenhagen, Denmark

**DOI:** 10.3201/eid1310.070503

**Published:** 2007-10

**Authors:** Mette Damkjær Bartels, Kit Boye, Anders Rhod Larsen, Robert Skov, Henrik Westh

**Affiliations:** * Hvidovre Hospital, Hvidovre, Denmark; † Statens Serum Institut, Copenhagen, Denmark

**Keywords:** MRSA, Panton-Valentine leukocidin, MLST, *spa* typing, Staphylococcus aureus, Community-onset MRSA, SCC*mec*, USA300, PFGE, Nursing home, research

## Abstract

In Copenhagen, methicillin-resistant *Staphylococcus aureus* (MRSA) accounted for <15 isolates per year during 1980–2002. However, since 2003 an epidemic increase has been observed, with 33 MRSA cases in 2003 and 110 in 2004. We analyzed these 143 cases epidemiologically and characterized isolates by pulsed-field gel electrophoresis, *Staphylococcus* protein A (*spa*) typing, multilocus sequence typing, staphylococcal chromosome cassette (SCC) *mec* typing, and detection of Panton-Valentine leukocidin (PVL) genes. Seventy-one percent of cases were community-onset MRSA (CO-MRSA); of these, 36% had no identified risk factors. We identified 29 *spa* types (t) and 16 sequence types (STs) belonging to 8 clonal complexes and 3 ST singletons. The most common clonal types were t024/ST8-IV, t019/ST30-IV, t044/ST80-IV, and t008/ST8-IV (USA300). A total of 86% of isolates harbored SCC*mec* IV, and 44% had PVL. Skin and soft tissue infections dominated. CO-MRSA with diverse genetic backgrounds is rapidly emerging in a low MRSA prevalence area.

## Abstract 2

Community-onset MRSA with diverse genetic backgrounds is rapidly emerging in this previously low MRSA prevalence area.

For many years, methicillin-resistant *Staphylococcus aureus* (MRSA) has been a serious and common nosocomial pathogen in hospitals outside the Nordic countries and the Netherlands (*1*). Community-onset MRSA (CO-MRSA) was first reported in Western Australia in the early 1990s (*2*) and has, especially during the past 5 years, emerged as a global problem (*1*,*3*). CO-MRSA in the United States has mostly been caused by the Panton-Valentine leukocidin (PVL)–positive clones USA400 (sequence type [ST]1) and more recently by USA300 (ST8) (*1*,*4*–*7*). In Europe the increase in CO-MRSA has mostly been attributed to the PVL-positive ST80 clone (*3*,*8*). In Denmark, since 1980, MRSA has accounted for <100 MRSA isolates per year nationwide (www.danmap.org/pdffiles/danmap_2005.pdf). At our laboratory, MRSA has been isolated from <15 patients per year until 2002. Approximately half of the MRSA cases found in Denmark during these years were imported, i.e., through patients transferred from foreign hospitals (*9*).

Since 2003 the number of MRSA-positive persons increased both nationally and in Copenhagen. Nationally, the number increased from 50–105 new cases per year to 243 in 2003, 549 in 2004, and 864 in 2005. At Hvidovre Hospital, we found MRSA in 5 persons in 2001, 14 in 2002, 33 in 2003, 110 in 2004, and 170 in 2005. We describe the epidemiology of the emergence of MRSA in Copenhagen in 2003 and 2004 and characterize the genetic background of the isolates.

## Materials and Methods

### Setting

The Department of Clinical Microbiology, Hvidovre Hospital, services 4 of the 5 hospitals in Copenhagen and receives all microbiology samples from general practice in the Copenhagen and Frederiksberg Municipality (population 597,000). The total number of swabs received in the laboratory decreased slightly from 60,245 in 2000 to 55,223 in 2004. The principles for MRSA treatment, isolation of all hospitalized MRSA patients and hospital and household screening, were not changed before or during the study period. The only change was the introduction of new molecular typing methods.

### Patient Data

Upon identification of an MRSA isolate, the patient’s attending physician was contacted to ensure correct antimicrobial chemotherapy and isolation procedures. The patient’s physician was interviewed about the patient to define types of infections, identify risk factors, and establish epidemiologic links between patients. Most interviews were followed up by 1–5 more contacts. All interview data were registered prospectively in case-report forms and entered into the patient record files of our laboratory information system. The National Health Care Database (NHCD) was used to check for hospital admissions and ambulatory healthcare visits in Danish hospitals within the previous 2 years. These data enabled us to identify whether MRSA patients had been hospitalized in the same ward during the same period. The patients’ home addresses, household members, and their general practitioners were identified in the NHCD. The patients were divided into 4 groups of acquisition mode of MRSA (*8*): 1) hospital-acquired (HA)–MRSA, MRSA isolated in a sample taken >48 h after admission or known exposure in a Danish hospital (domestic HA-MRSA) or a hospital abroad (imported HA-MRSA); 2) community-onset MRSA healthcare-associated (CO-HCA), MRSA isolated in a sample taken <48 hours after hospital admission or in general practice, with hospitalization and/or outpatient treatment in the previous year, having close contact to a person with HA-MRSA, residing in a nursing home, receiving in-home help or home nursing, being on dialysis, having cancer or diabetes, or being a drug abuser; 3) CO-MRSA community risk (CO-CR), MRSA isolated in a sample taken <48 hours after hospital admission or in general practice and having close contact to a person with CO-MRSA or having recently traveled to a country with high MRSA endemicity (no known contact with healthcare); and 4) CO-MRSA with no known risk factors (CO-NR), MRSA isolated in a sample taken <48 hours after hospital admission or in general practice, with no healthcare association, no known community risk, and no travel risk.

### MRSA Isolates and Antimicrobial Susceptibility Testing

*S. aureus* isolates were identified by positive Staphaurex (Remel Europe Ltd., Dartford, UK) result and a positive coagulase test result. Only the first MRSA isolate from each patient was included in our study, for a total of 143 isolates in 2003 and 2004.

Susceptibility testing was performed on Isosensitest agar by the disk-diffusion method (antibiotic disks; Oxoid, Basingstoke, UK) according to recommendations of the Swedish Reference Group for Antibiotics (www.srga.org). Isolates were screened for resistance to methicillin by a cefoxitin 10-μg disk (*10*). Isolates were further tested against penicillin, cefuroxime, erythromycin, clindamycin, gentamicin, vancomycin, fucidic acid, rifampin, tetracycline, and moxifloxacin. Inducible clindamycin resistance was examined by using the double-disk method (*11*). All MRSA isolates were confirmed *mec*A positive by PCR (*12*). Multidrug-resistant MRSA was defined as an isolate resistant to >3 non–β-lactam antimicrobial agents.

### Typing Methods

*Sma*I macrorestriction profiles were performed according to the HARMONY protocol (*13*) and analyzed with Bionumerics version 4.5 software (Applied Maths, Kortrijk, Belgium). Concatamerized phage λ DNA (New England Biolabs, Ipswich, MA, USA) was loaded in every sixth lane as a molecular weight standard to normalize the individual gels, and *Sma*I-digested *S. aureus* NCTC 8325 was used to normalize the gels to each other. Only DNA fragments in the range of the λ ladder (50–1,000 kb) were included in the analysis. Pulsed-field gel electrophoresis (PFGE) clusters were identified on an UPGMA (unweighted pair-group method with arithmetic mean) dendrogram based on Dice coefficients, where optimization and band position tolerance were set at 1% and 2%, respectively. A similarity coefficient of 80% was selected to define the clusters. Reference strains used for PFGE were USA100–1100 (Centers for Disease Control and Prevention, Atlanta, GA, USA) and UK-MRSA 1, 3, 15, and 16 (HARMONY collection).

Sequencing of the staphylococcal protein A gene (*spa* typing) and multilocus sequence typing (MLST) were performed as previously described (*14*,*15*), except that PCR products were enzymatically purified with exonuclease I (New England Biolabs) and shrimp alkaline phosphatase (Amersham Biosciences, Piscataway, NJ, USA) before *spa* sequencing. Sequence reactions were performed on both DNA strands and analyzed on an ABI Prism 3100 (Applied Biosystems, Foster City, CA, USA). For PCR and sequencing of the *spa* gene, primers 1113F and 1496R were used. Designation of *spa* type was conducted by using the Ridom StaphType program (www.ridom.de) (*16*). MLST was performed on 57 (40%) of the 143 isolates, representing all *spa* types, and on every fifth randomly chosen isolate if the *spa* type was t024, t019, t044, or t008. STs and clonal complexes (CCs) were assigned through the MLST database (www.mlst.net).

Staphylococcal chromosome cassette (SCC) *mec* types were determined by an in-house multiplex PCR strategy in which types I–V were identified (*17*). Presence of PVL determinants was detected by PCR by using primers that overlap with previously published primer sequences (*18*,*19*). Primer sequences were forward 5′-TAG-GTA-AAA-TGT-CTG-GAC-ATG-3′ and reverse 5′-GCA-TCA-AST-GTA-TTG-GAT-AGC-3′.

## Results

### Patient and Infection Characteristics

In 2003 and 2004, 33 and 110 new cases of MRSA, respectively, were found. In 126 cases (88%) MRSA was from infection, and in 17 cases (12%) MRSA was found through screening of close family or hospital contacts to known MRSA patients.

Of the 143 MRSA cases, 42 (29%) were HA; 5 (3%) were imported, and 101 (71%) were CO. In 36 of the 101 CO-MRSA cases, the patient had a healthcare risk (CO-HCA), primarily due to hospitalization during the past year (21 cases) or residency in a nursing home (14 cases). Seven of the 36 patients were identified during the first 48 hours of hospitalization. For 30 patients a community risk (CO-CR) was identified, and for 35, no risk factors (CO-NR) were identified.

MRSA was found in samples from 50 patients during their hospitalization. Seventeen of these patients were in single-bed rooms; for those in shared rooms, we had no data on possible roommates for 5 case-patients, and the roommates of 3 case-patients were not screened. For 21 case-patients, MRSA screening of roommates was performed. Screening showed 4 MRSA-positive roommates with MRSA strains of the same *spa* type as that of the index patient (3 t024, 1 t015). In 1 intensive care unit case, transmission presumably occurred from staff to a patient in an adjoining room, as the *spa* type found (t003) was unique among our isolates.

Transmission among the 93 patients that were not hospitalized was more common. Of these, 11 resided in nursing homes; 5 were in the same nursing home. Another 14 patients lived alone. For 15 patients it was not possible retrospectively to determine whether anyone had lived with the patient (in most cases the index patient had died). In the households of 11 index patients, another 13 persons were found to be MRSA positive. The *spa* type most often found in family members was t019/ST30. In 11 families, all household members were MRSA negative. Household screening was not performed for 18 families.

Most of our MRSA cases were in the Amager region of Copenhagen. The 2-year incidence of CO-MRSA (HCA included) in Amager was 40:100,000 compared with 12:100,000 in the rest of Copenhagen.

Skin and soft tissue infections dominated (91 [64%] of 143 cases), followed by 17 urinary tract infections (UTIs, 12%), 9 deep-seated abscesses (6%), 5 lower respiratory tract infections (LRTIs, 3%), and 4 cases of septicemia (3%) (Table 1; Figure). Patients with HA-MRSA and CO-HCA MRSA had similar median ages: 78 (range 28–94 years) and 82 years (6–95 years), respectively. The patients with CO-NR MRSA and CO-CR MRSA had median ages of 32 (range 0–90 years) and 27 years (1–74 years), respectively (Table 1). The HA-MRSA and CO-HCA MRSA patients as well as the CO-NR MRSA and CO-CR MRSA patients were in many aspects similar and will be described as 1 group each.

**Table 1:** Demographics, infection types, and distribution of MRSA types in 143 cases of MRSA*

	Community onset, community risk	Community onset, no risk	Hospital acquired in Denmark	Community onset, healthcare associated	Imported
No. cases	30	35	37	36	
Male, %	67	46	57	42	
Median age, y (range)	27 (1–74)	32 (0–90)	80 (28–94)	82 (6–95)	
Carrier, no.	8	1	2	2	4†
Type of infection, no.					
SSTI	22	29	17	23	
Blood	0	0	2	2	
Deep-seated abscess, no.	0	1	5	3	
UTI	0	4	8	5	
LRTI	0	0	3	1	1†
Four most common CCs (87% of all isolates)					
CC 8, %	23	34	81	69	
t008, no.	3	7	0	2	
t024, no.	3	5	28	20	
CC 80, %	20	34	3	0	
CC 30, %	50	6	0	0	
CC 5, %	3	11	5	8	
Other MLST types,%	4	15	11	23	
PVL positive, %	83	80	8	17	
SCC*mec* IV, %	93	89	89	81	

*MRSA, methicillin-resistant *Staphylococcus aureus*; SSTI, skin and soft tissue infection; UTI, urinary tract infection; LRTI, lower respiratory tract infection; CC, clonal complex; MLST, multilocus sequence typing; PVL, Panton-Valentine leukocidin; SCC, staphylococcal chromosome cassette. †A t037/ST239-III was imported from Greece, where this MRSA is common (*20*); ST239 is closely related to ST8 and is the well-known pandemic Brazilian/Hungarian clone (*21*); t041/ST111 was imported from an Italian hospital; t003/ST225-II (New York clone) was imported from Germany; t354/ST22-IV, a variant of EMRSA15, was imported from Lanzarote (Spain); and t067/ST125-I was imported from Spain.

**Figure:**
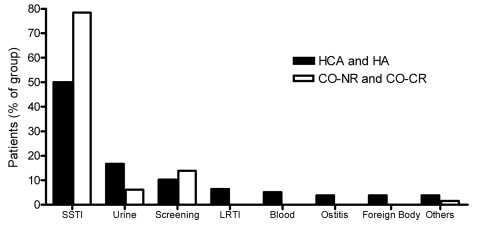
Distribution of infection types in community-onset methicillin-resistant *Staphylococcus aureus* (MRSA) and hospital-acquired (HA) MRSA. HCA, healthcare associated; CO-NR, community-onset MRSA with no identified risk factors; CO-CR,community-onset MRSA with community risk; SSTI, skin and soft tissue infection; LRTI, lower respiratory tract infection.

### Susceptibility Testing of Isolates

Resistance to macrolides was detected in 85 (59%) of 143 isolates and in all isolates of *spa* type t024 and 7 of 12 t008 isolates. Constitutive macrolide resistance was found in 48% of erythromycin-resistant isolates. The resistance profiles of the 11 multiresistant isolates are described in the Appendix Table (*3*,*8*).

#### HA and CO-HCA MRSA

Of 11 multidrug-resistant MRSA, 8 belonged to this group. Resistance to β-lactams only was found in 6 CO-HCA MRSA isolates (6 [8%] of 78) that belonged to 5 different CCs and contained either SCC*mec* IV or V.

#### CO-NR MRSA and CO-CR MRSA

Of 11 multidrug-resistant MRSA, 3 were in this group and 2 patients had no identified risk factors (t002/CC5 and t044/ST80). Of 65 isolates, 23 (35%) were resistant only to β-lactams, and all contained SCC*mec* IV. More data on resistance can be seen in the Appendix Table.

#### PFGE, *spa* Typing, and MLST

There was a strong overall match between the clustering based on PFGE dendrogram, *spa* type, ST, or CC of the analyzed isolates (Table 2; Appendix Figure). The sequence-based typing methods showed 16 STs and 29 *spa* types (Table 2). MRSA isolates belonged to 8 CCs; CC8 was the most frequent (Table 2). The most common ST was ST8, represented by 72 isolates. The dominant *spa* type was t024/ST8, which accounted for 56 (39%) of 143 isolates. Other common MRSA were *spa* type t019/ST30 (17 isolates), *spa* type t044/ST80 (16 isolates), and *spa* type t008/ST8 (12 isolates). The 5 imported MRSA belonged to CC5 (2 isolates), CC 8 (t037/ST239-III), CC 22, and ST111. One CC1 strain was placed far from the other 2 CC1 isolates. The first isolate was ST748, t127, PVL negative, and SCC*mec* V; the other 2 were ST1, t175, PVL positive, and SCC*mec* IV (USA400-like). PFGE could not discriminate between MRSA t008, PVL-positive isolates, and t024 PVL-negative isolates but intermingled these isolates.

**Table 2:** Genetic backgrounds and epidemiologic information on MRSA isolates*

CC	Multilocus ST	*Spa* types	Isolates, no. (total in CC)	No. PVL-positive isolates	SCC*mec* types (no. isolates)	HA/HCA MRSA or CR/NR MRSA % in the CC
CC1	ST1 ST748†	t175 t127	2 1 (3)	2 0	IV V	33/67
CC5	ST5 ST125 ST225	t002 t067 t003	7 1 4 (12)	7 0 4	I (3), II (1), IV(3) I II	58/42
CC8	ST8 ST239	t024, t008, t430 t064, t351 t037	56/12/2 1/1 3 (75)	12 (t008) 0 0	IV IV III (2), NT (1)	74/26
CC15	ST15	t084	1	0	V	100/0
CC22	ST22 ST737†	t005, t022, t354 t541 t542	2/1/1 1 1 (6)	0 0 0	IV, NT (t022) IV IV	50/50
CC30	ST30	t019	17	17	IV	0/100
CC45	ST45	t015, t026, t065 t116, t350	2/1/1 1/1 (6)	0 0	IV, V (t026 and t116)	100/0
CC97	ST97	t359	1	1	V	100/0
Singleton	ST80	t044, t376, t455	16/2/1 (19)	19	IV	5/95
Singleton	ST111	t041	1	0	I	100/0
Singleton	ST152	t355	1	1	V	0/100
Total			142	63		

*MRSA, methicillin-resistant *Staphylococcus aureus*; CC, clonal complex; ST, sequence type; PVL, Panton-Valentine leukocidin; SCC, staphylococcal chromosome cassette; HA/HCA/CR/NR, hospital acquired/healthcare associated/community risk/no risk. One isolate could only be typed by *spa* and multilocus sequence typing and is not included in the table. †New ST.

#### HA and CO-HCA MRSA

The 78 isolates (42 HA and 36 CO-HCA MRSA) represented 20 different *spa* types and 1 nontypeable isolate. A total of 48 (86%) of the 56 *spa* t024 were in this group. The patients were predominately elderly persons living in nursing homes or receiving home-care nursing. Most isolates belonged to CC8 (72%) (Table 2). Thirty-seven of the HA-MRSA isolates were considered Danish nosocomial isolates (domestic HA-MRSA), and 5 were from patients transferred from foreign hospitals (imported HA-MRSA).

#### CO-NR MRSA and CO-CR MRSA

The 65 isolates (35 CO-NR-MRSA and 30 CO-CR MRSA) represented 12 different *spa* types. All of the 17 t019/ST30, 15 of the 16 t044/ST80, and 10 of 12 of the t008/ST8 were found in this group. Only 8 patients with t024 were considered to have MRSA of CO-NR or CO-CR origin. Twenty-four cases were associated with household outbreaks, and transmission was suspected between children in 2 kindergarten classes.

### Distribution of SCC*mec* Types and Prevalence of PVL Genes

#### SCC*mec* Type

SCC*mec* types could be determined for 140 (98%) of 143 isolates. SCC*mec* type IV was found in 122 (86%) of the isolates. Eighty-one percent of the HA and CO-HCA MRSA and 91% of the CO-NR/CO-CR isolates harbored SCC*mec* IV. The remaining isolates were represented by 5 type I (3.5%), 5 type II (3.5%), 2 type III (1.5%), 6 type V (4%), and 3 nontypeable (NT) (2%). Five CCs contained >1 SCC*mec* type: CC1 had IV and V; CC5 had I, II and IV; CC8 had III, IV, and NT; CC22 had IV and NT; and CC 45 had IV and V (Table 2).

#### PVL

Sixty-three (44%) MRSA isolates carried the PVL genes. Twenty-nine (97%) of 30 abscesses were caused by PVL-positive MRSA. PVL-positive isolates were also seen in 19 patients with chronic wounds, 8 MRSA carriers, 4 patients with UTI, 1 patient with LRTI, 1 with ear infection, and 1 with pus from a gallbladder. *spa* types and PVL were clearly correlated: all t019/ST30, t044/ST80, t008/ST8, t002/ST5, and t003/ST5 were positive for the PVL genes. The remaining PVL-positive isolates were found in the following *spa* types: t175 (2/2), t355 (1/1), t359 (1/1), t376 (2/2), and t455 (1/1). None of the 56 *spa* t024 carried the PVL genes (Table 2).

#### HA and CO-HCA MRSA

Ten (13%) of the 78 isolates were PVL-positive. Three of them were part of a hospital outbreak with *spa* t003 imported in a patient from Germany.

#### C0-NR MRSA and CO-CR MRSA

Fifty-three (82%) PVL-positive isolates were found in the 65 strains in this group, 28 from patients with CO-NR MRSA and 25 from patients with CO-CR MRSA. PVL was present in 82% of CO-NR and CO-CR isolates, the isolates that we regard as truly community-acquired MRSA isolates.

## Discussion

The high degree of genetic diversity (in both MLST background and SCC*mec* cassettes) found among MRSA isolates from a low-prevalence area is of global public health concern. The number of MRSA isolates doubled in <1 year in our area. Had the spread continued unhindered, MRSA would very rapidly have become a major source of illness as well as a healthcare financial concern for our hospitals and in the community. Although national guidelines to prevent MRSA transmission were first established in November 2006, an intervention program was introduced in Copenhagen in September 2005. We believe that our intervention program has had some effect: the number of cases in 2005 (170) increased to only 189 in 2006. While the current increase in CO-MRSA in the United States is predominantly by only 1 clone, USA300, we found 29 different *spa* types and 16 different STs in an area of <50 square miles. This finding suggests that the diversity has been caused not only by the spread of clones but also by the dissemination of the more mobile SCC*mec* elements IV and, to a lesser degree, V into methicillin-sensitive *S. aureus* (MSSA) (*22*). Typing MRSA with different methods gave additional information. The use of *spa* typing and PVL allowed the differentiation of MLST and PFGE identical isolates, and SCC*mec* typing could explain why MLST identical isolates had different PFGE patterns.

Our dominant clone, *spa* t024/ST8-IV, accounted for 39% of all isolates. The MRSA *spa* t024 clone is new in Denmark (*8*) and has been only sporadically reported, as MRSA or MSSA, to the SeqNet *spa* database (www.spaserver.ridom.de). The t024 clone was PVL negative but carried SCC*mec* IV, as did the typical PVL-positive community clones t044/ST80, t019/ST30, and t008/ST8. t024 belongs to CC8, as did the archaic clone that caused hospital outbreaks in Denmark in the late 1960s and early 1970s (*23*). The PFGE pattern of t024 is almost identical to that of t008 (USA300) but can be differentiated by being PVL negative and having a *spa* type that has lost 24 consecutive bases (1 repeat). The PVL phenotype of t024 CC8 strains was more like HA-MRSA around the globe. t024 was predominantly associated with nursing home outbreaks and home care nursing in the same area of Copenhagen as the nursing homes. Hospitalization of these patients led to small MRSA outbreaks. Our infection control team trained staff in the affected nursing homes in infection control. Isolation procedures in nursing homes were modified, compared with hospital regimes, to respect the fact that the patients were living in their own home. The nursing home staff were predominantly social workers with rudimentary infection control education. This, combined with the fact that many patients were not eligible for eradication treatment because of chronic wounds, could explain some of the difficulties in limiting the spread in nursing homes.

On the basis of *spa* typing, we have seen some evolution of the t024 clone. *spa* type t430 has evolved from t024 by the loss of 24 consecutive bases. The 2 patients with t430 were identified late in the t024 outbreak, and we find it more likely that a deletion occurred in t024 rather than that a new MRSA clone was introduced or that the SCC*mec* was transmitted to MSSA. In support of this view, samples from 1 patient taken the same day had t024 (axilla sample) and t430 (nose sample) (data not shown).

Some of the internationally well-known CO-MRSA clones were among our isolates (*3*,*24*). Most of the t008/ST8-IV could be identified as MRSA USA300–0114 genotype on the basis of PFGE typing (data not shown) (*25*). This finding is very troublesome because USA300 has evolved as the most rapidly spreading CO-MRSA in the United States and has become a dominant HA-MRSA in some hospitals (*24*). Special attention must be taken to control the spread of this clone. Sixteen (11%) isolates were t044/ST80-IV, which is a common CO-MRSA in Europe (*3*,*26*). This was a much more prevalent CO-MRSA clone in a national study of 81 Danish MRSA isolates from 2001, where 44% belonged to ST80-IV (*8*). We found that t019/ST30-IV was the most common CO-MRSA (17 patients, 12%). The t019/ST30-IV clone is known as the Southwest Pacific clone, and many of our cases had a geographic link to the Far East (data not shown). t019 was PVL positive, generally susceptible to all non–β-lactams, and caused severe abscesses. The PVL-positive MRSA clones were almost never associated with hospital outbreaks probably because these patients with skin and soft tissue infections were rapidly discharged.

Our findings of high prevalence of PVL-positive isolates (44%) are not surprising and are consistent with data from other studies (*27*–*29*) in which PVL is associated with skin and soft tissue infections, commonly described in CO-MRSA. Eighty-two percent of the CO-NR/CO-CR isolates contained the PVL genes, compared with only 13% of the HA/CO-HCA MRSA. We observed an age difference with a median age of 32 years (0–90 years) in the NR/CR group compared with 82 years (6–95 years) in the HCA group. These findings might indicate that the first group consists of patients with true community-onset MRSA and the second group is closely related to our HA-MRSA. Current definitions are becoming of decreasing usefulness as typical CO-MRSA strains invade hospitals.

A possible explanation for the increase in CO-MRSA could be a rise in outpatient antimicrobial drug use. Compared with other European countries (average 20 defined daily dose [DDD]/1,000 inhabitants/day) and the United States (26 DDD/1,000 inhabitants/day), Denmark had a low level of antimicrobial drug use in 2004 (14 DDD/1,000 inhabitants/day) (*30*). Although the use of systemic antimicrobial agents in Denmark has increased 14.7% from 2000 through 2004 (www.dfvf.dk/files/filer/zoonosecentret/publikationer/danmap/danmap_2004.pdf), it is unlikely that this is the only reason for the rise in MRSA.

Although we found a high number of CO-MRSA, our assessment of risk factors has some limitations. Having the data of all hospital admissions and outpatient hospital visits from the NHCD makes the division into the categories of HA-MRSA and CO-MRSA almost certain. However, because most of the patients were not directly interviewed about risk factors, some data might have been missed. Therefore, some of the CO-NR MRSA might actually be CO-CR or CO-HCA MRSA.

Previous guidelines used in Denmark for controlling MRSA in the hospitals by screening patients transferred from hospitals abroad are not sufficient to address today’s CO-MRSA problems. In November 2006, the Danish National Health Board published national guidelines for preventing MRSA spread (www.sst.dk/publ/publ2006/cff/mrsa/vejl_mrsa.pdf). These guidelines introduce new approaches to limit the spread of MRSA in hospitals and in the community, where isolation of patients is not an option. In some hospitals we now have introduced admittance MRSA screening of all patients who have wounds or urinary catheters and we are isolating all residents from MRSA-endemic nursing homes. The hospital infection control teams see all patients with MRSA. In the community we have improved the screening of close contacts of MRSA patients and initiated a task force that visits patients at home and plans the eradication of MRSA carriage for all persons in the household. Our initial regime is 5 days’ treatment with 4% chlorhexidine body wash once a day and 2% mupirocin nasal ointment 3× each day combined with washing of towels and bed linen and improved housekeeping. For some patients the treatment needs to be repeated (*31*), and patients with MRSA carriage that is difficult to eradicate are sometimes treated with systemic antimicrobial agents (*32*). Unfortunately, not all patients are decolonized by this procedure, and the long-term effect of eradication treatment, including the possible development of resistance to the antimicrobial agents used, is not well documented (*33*). Long-term studies on new and more effective methods to eradicate MRSA carriage are needed.

The rapid increase and genetic diversity of CO-MRSA in a country with low prevalence of HA-MRSA are of great concern, as these CO-MRSA are sporadically appearing in our hospitals. Especially worrisome is the discovery that MRSA USA300 genotype is in our community, as this MRSA has recently shown its epidemic potential in both the US community and US hospitals (*24*). MRSA isolation rates are increasing in other Nordic countries (*34*,*35*), but we need more data on CO-MRSA from countries in which classic HA-MRSA dominate. The current search-and-destroy policies need to be updated, refined, and intensified if Denmark hopes to remain a country with low MRSA prevalence.

## Supplementary Material

Appendix Figure Cluster analysis by pulsed-field gel electrophoresis (PFGE). Data on spa type, staphylococcal chromosome cassette (SCC) mec type, Panton-Valentine leukocidin (PVL), and sequence type (ST) are included. Two isolates (t024 and t359) could not be typed by PFGE.

Appendix Table Resistance patterns of 143 multiresistant isolates of Staphylococcus aureus*
