# Supplementary figures and images for: Rapid Increase of Genetically Diverse Methicillin-Resistant Staphylococcus aureus, Copenhagen, Denmark
Source: Emerg Infect Dis. 2007 Oct;13(10):1533–40. doi: 10.3201/eid1310.070503 (PMC2851516; doi:10.3201/eid1310.070503)

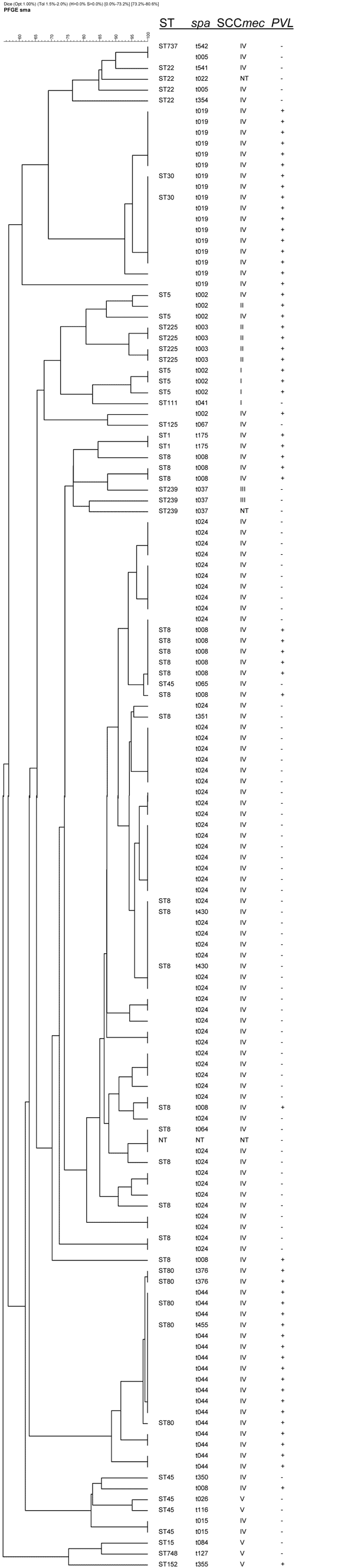

Supplement: Appendix Figure — Cluster analysis by pulsed-field gel electrophoresis (PFGE). Data on spa type, staphylococcal chromosome cassette (SCC) mec type, Panton-Valentine leukocidin (PVL), and sequence type (ST) are included. Two isolates (t024 and t359) could not be typed by PFGE. [file 07-0503_appF-s1.gif]
