# Supplementary material for: Rapid Increase of Genetically Diverse Methicillin-Resistant Staphylococcus aureus, Copenhagen, Denmark
Source: Emerg Infect Dis. 2007 Oct;13(10):1533–40. doi: 10.3201/eid1310.070503 (PMC2851516; doi:10.3201/eid1310.070503)
Supplement: Appendix Table — Resistance patterns of 143 multiresistant isolates of Staphylococcus aureus* [file 07-0503_appT-s2.pdf]

Appendix Table. Resistance patterns of 143 multiresistant isolates of *Staphylococcus aureus**

CC/singleton	Beta	E,C	E,C,F	E,C,G	E,C,M	E,C,T	F	G	M	M,T	T	F,T	E,C,F,M,T	E,C,F,T	E,C,G,M,T	E,C,M,T	E,C,G,M	Total
CC1	3																	3
CC5		1	3		4		1			1						2		12
CC8	4	36	3	2	19	4	1				1		1		1	2	1	74
CC15	1																	1
CC22	4				1				1									6
CC30	16											1						17
CC45			1					2			2					1		6
CC97	1																	1
ST80							1					16		2				19
ST111																	1	1
ST152								1										1
NT											1							1
Total	29	37	7	2	24	4	3	3	1	1	4	17	1	2	1	5	2	143
HA and CO-HCA	6	28	3	2	20	3	1	2	0	1	4	0	1	1	0	4	2	78
CO-NR and CO-CR	23	9	4	0	4	1	2	1	1	0	0	17	0	1	1	1	0	65

*Numbers in each column are the number of isolates with the specific resistance profile. In the rows the resistance pattern in the clonal complex (CC)/sequence type (ST) singletons and in hospital-acquired/healthcare-associated/community risk/no risk (HA/CO-HCA) MRSA and community onset–no health care association–community risk (CO-NR/CO-CR) MRSA are presented. Beta, resistant only to beta-lactams; E, erythromycin; C, clindamycin; F, fucidic acid; G, gentamicin; M, moxifloxacin; T, tetracycline; NT, nontypeable.
